# Supplementary material for: Multiomic single cell sequencing identifies stemlike nature of mixed phenotype acute leukemia
Source: Nat Commun. 2024 Sep 18;15:8191. doi: 10.1038/s41467-024-52317-2 (PMC11411136; doi:10.1038/s41467-024-52317-2)
Supplement: Supplementary file 1 — Supplementary Information [file 41467_2024_52317_MOESM1_ESM.pdf]

a

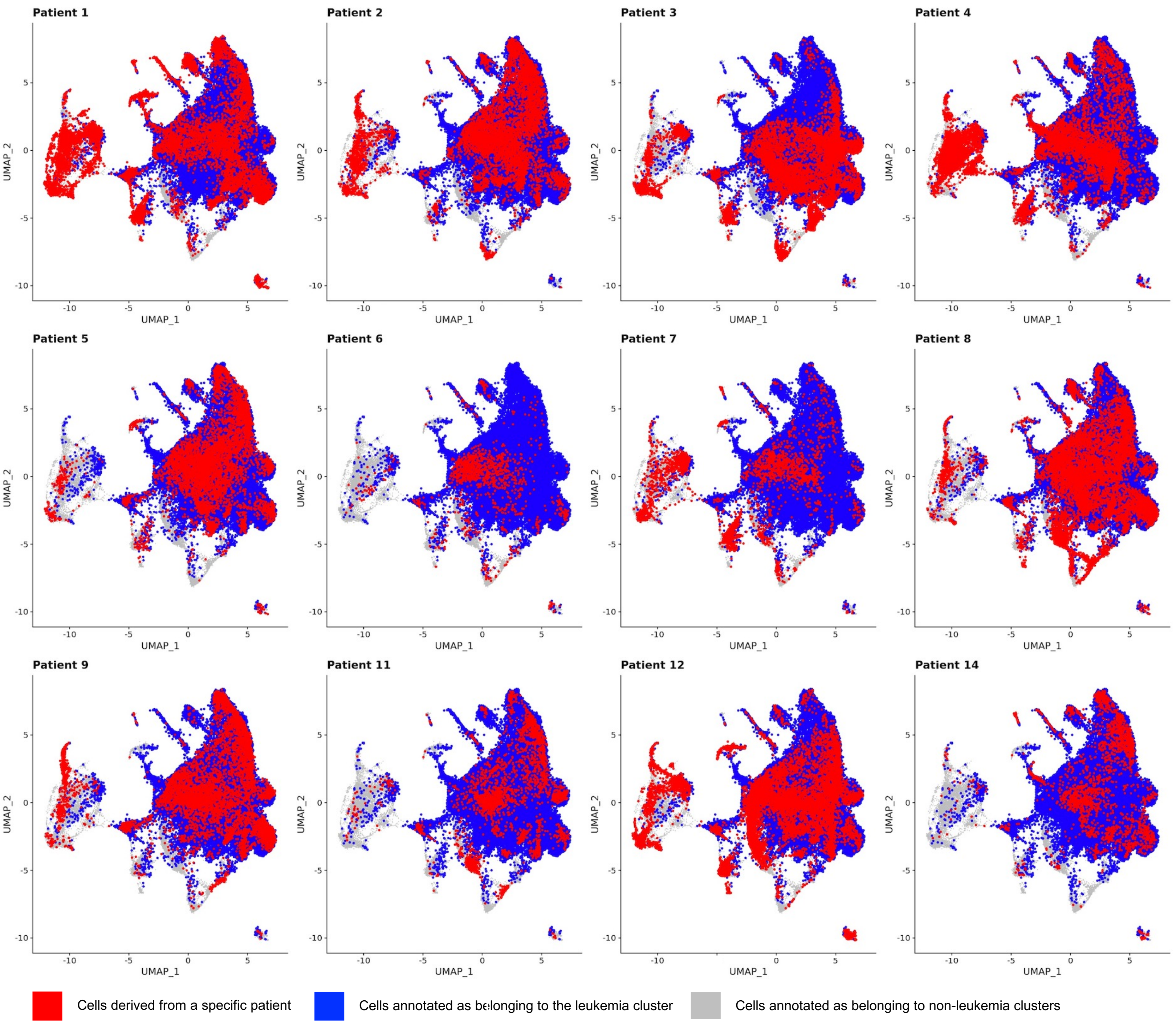

b

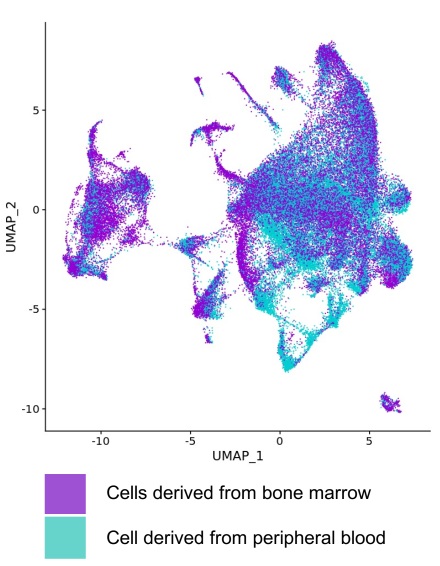

Supplementary Figure 1. RNA-derived UMAP from SC RNA+protein analysis of 71,579 cells from 12 patients. A. For each of the 12 panels, cells color-coded in red are derived from each of the 12 patients, cells color-coded in blue are in the leukemia cluster, and cells color-coded in grey are in non-leukemia clusters. All patients contributed to the leukemia cluster. B. Cells are color-coded based on whether the diagnostic sample was derived from bone marrow or peripheral blood. Source data for all panels are provided as a Source Data file.

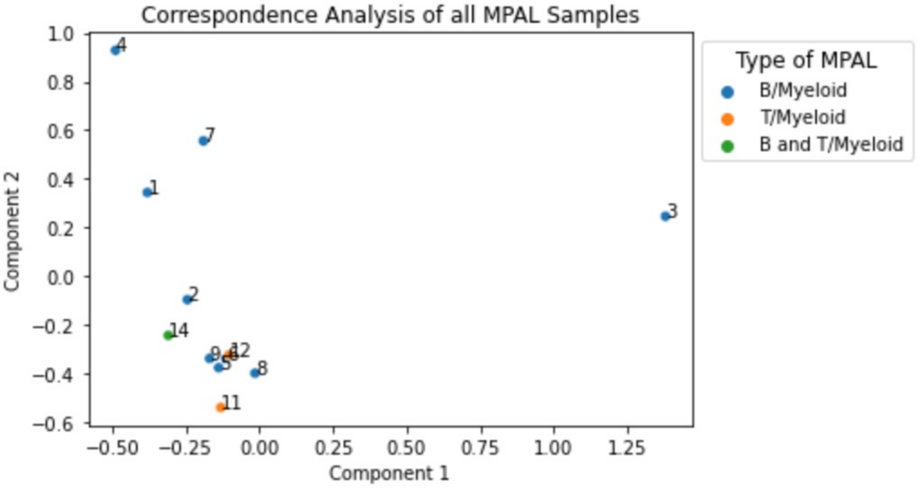

Supplementary Figure 2. Correspondence Analysis based on transcriptional data from 12 patients with MPAL. Each point represents an individual patient with patient numbers overlain. Points are color-coded based on immunophenotypic subtype. Source data are provided as a Source Data file.

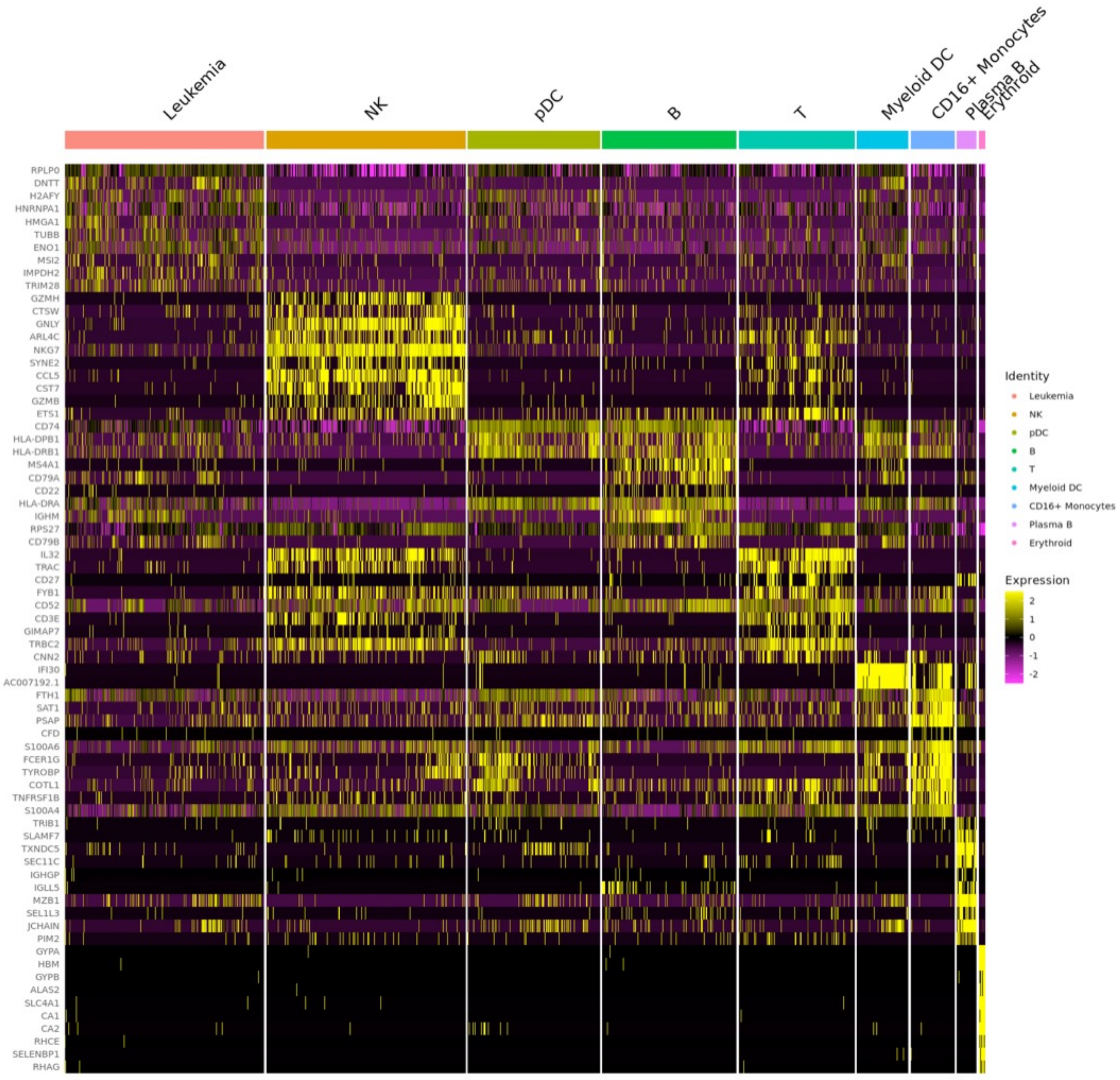

Supplementary Figure 3. Heatmap of scaled expression values for top ten most upregulated conserved genes for each transcriptionally defined cell type as identified in **Figure 1B**. Source data are provided as a Source Data file.

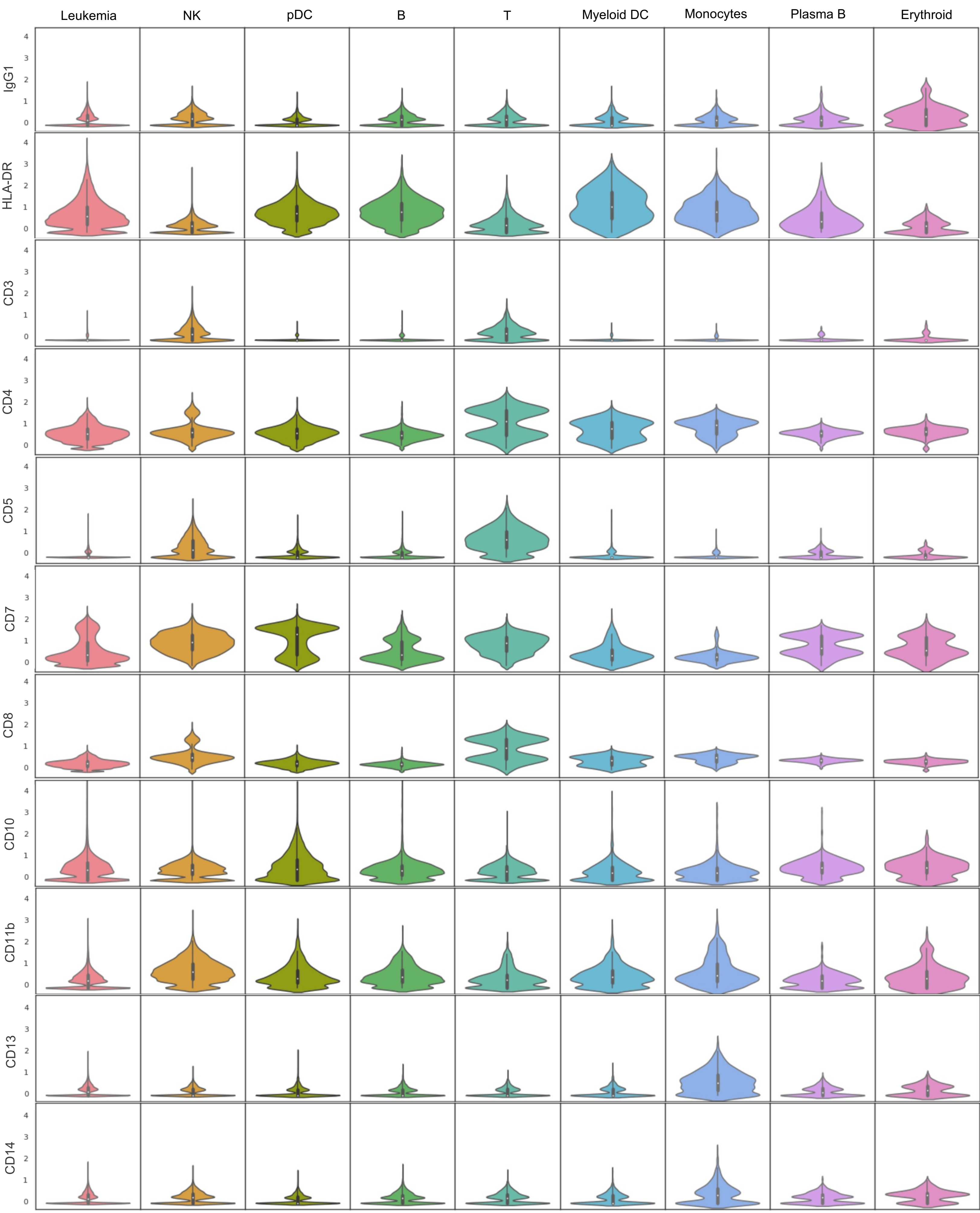

Leukemia
NK
pDC
B
T
Myeloid DC
Monocytes
Plasma B
Erythroid
IgG1
HLA-DR
CD3
CD4
CD5
CD7
CD8
CD10
CD11b
CD13
CD14
0
1
2
3
4

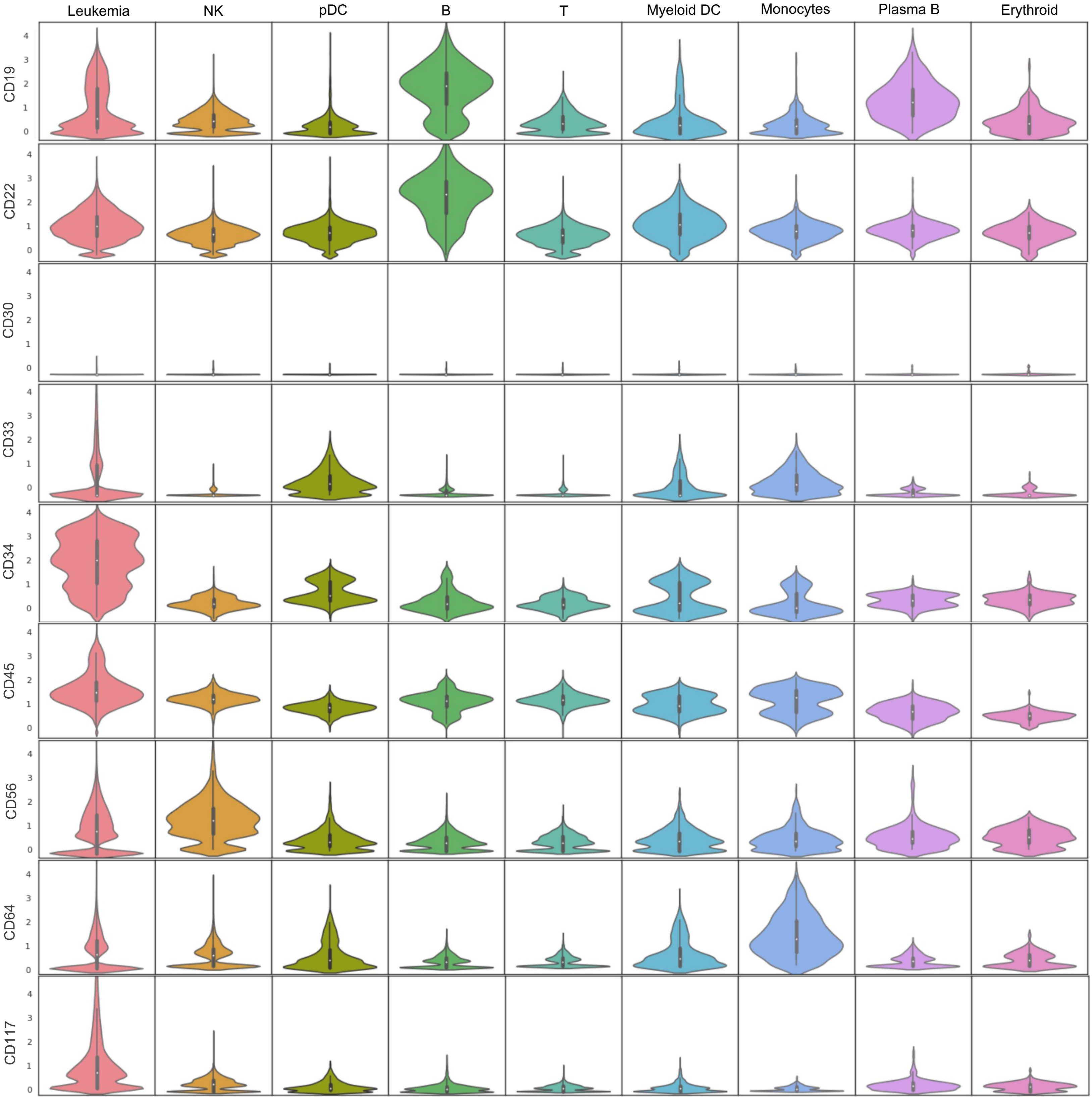

Supplementary Figure 4. Array of violin plots normalized cell-surface antibody expression from single-cell RNA+protein analysis of 71,579 cells from 12 patients with MPAL. Columns represent transcriptionally-defined cell types, and rows represent normalized cell-surface antibody expression. Cell types are annotated based on transcriptional data using a combination of scType and clustifyr, with immature populations collapsed into a common 'leukemia' cluster. Source data are provided as a Source Data file.

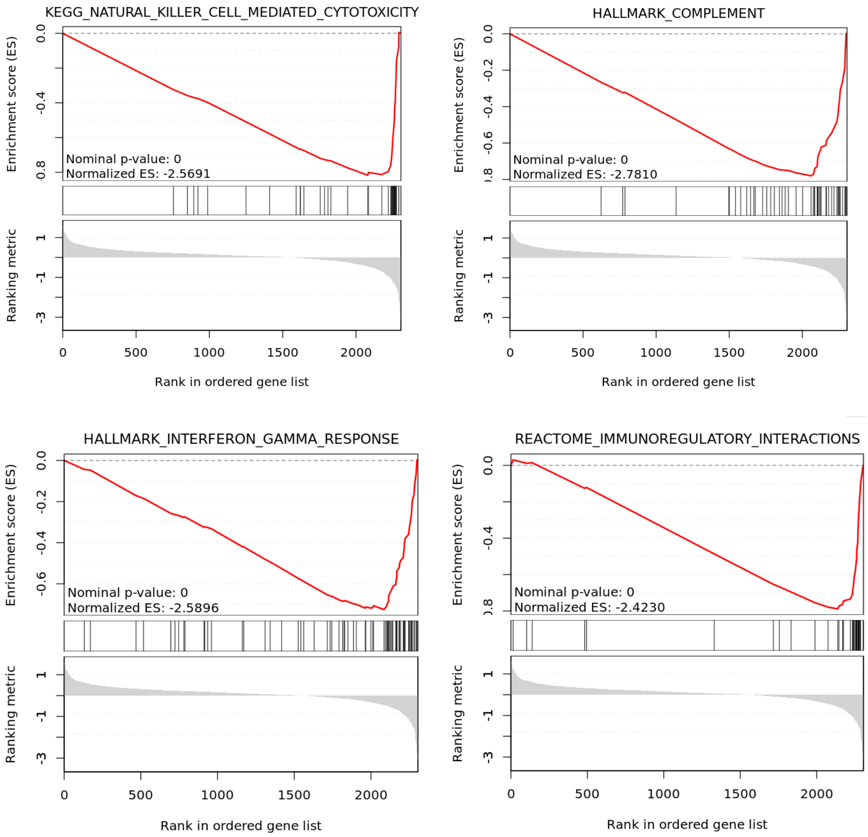

Supplementary Figure 5. Gene Set Expression Analysis (GSEA) of all cells transcriptionally annotated as Leukemia vs non-Leukemia across 12 patients with MPAL.

Enrichment profile and ranking metric score for four example negatively enriched gene sets, all associated with immune signaling. The GSEA analysis employs a one-sided permutation-based test to determine the significance of gene set enrichment, with raw p-values adjusted for multiple testing using the Benjamini-Hochberg procedure to control the false discovery rate (FDR). Source data are provided as a Source Data file.

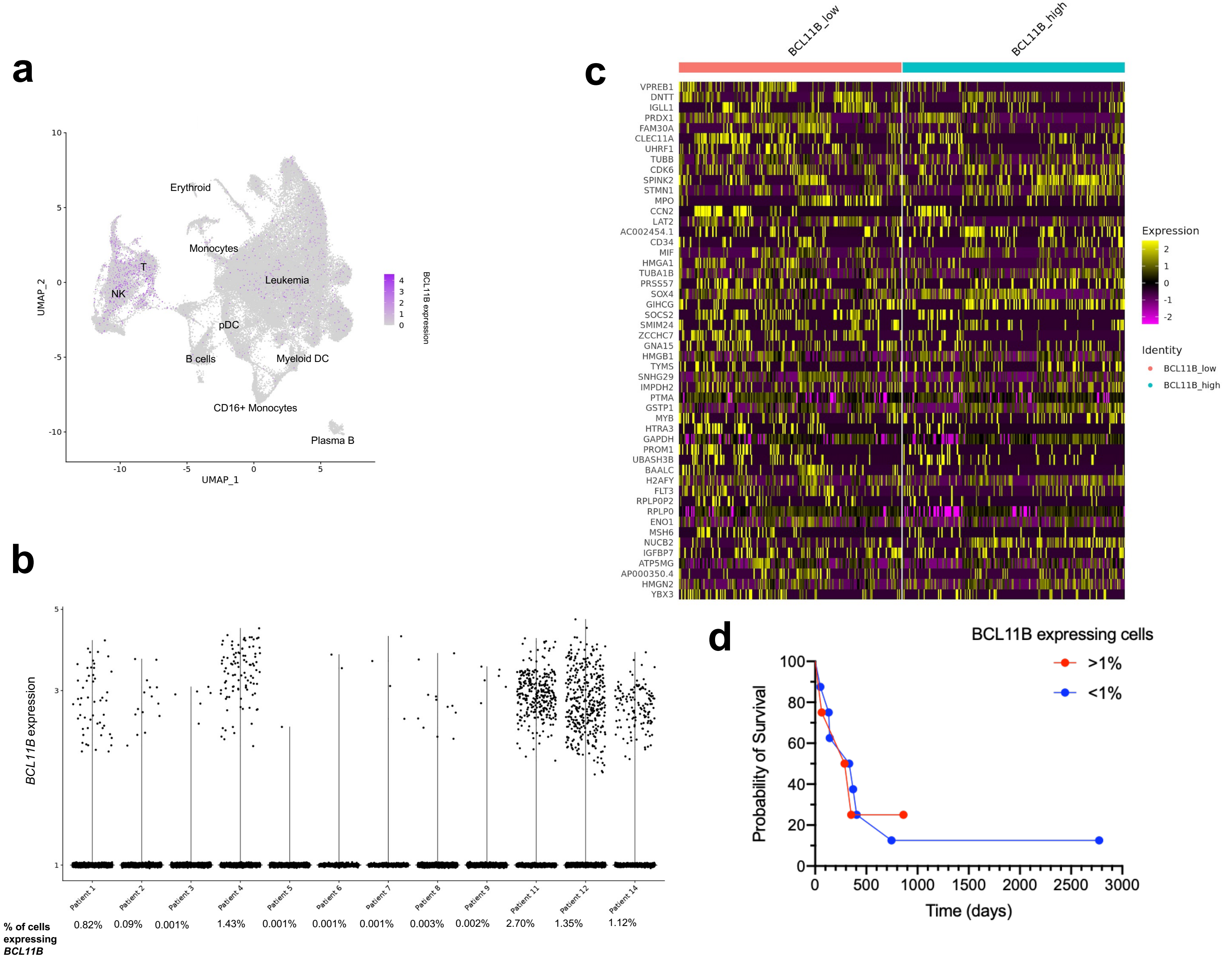

Supplementary Figure 6. *BCL11B* is overexpressed in <1% of blasts and does not associate with the shared MPAL gene expression signature or patient outcomes

A. RNA-derived UMAP from Figure 1B, derived from single-cell RNA analysis of 71,579 cells from 12 patients with MPAL. Cells are annotated in purple based on expression of *BCL11B.* In the common Leukemia cluster, 406 (0.76%) cells expressed *BCL11B.* B. Dot plot of *BCL11B* expression stratified by patient. The percent of total *BCL11B*-expressing cells is indicated below each bar C. Heatmap comparing gene expression of *BCL11B* expressing (vs non-expressing cells for the most upregulated genes in the common MPAL leukemia cluster. D. Kaplan-Meier estimate of overall survival stratified by *BCL11B* expression (expressed in <1% vs >1% of cells). Source data for all panels are provided as a Source Data file.

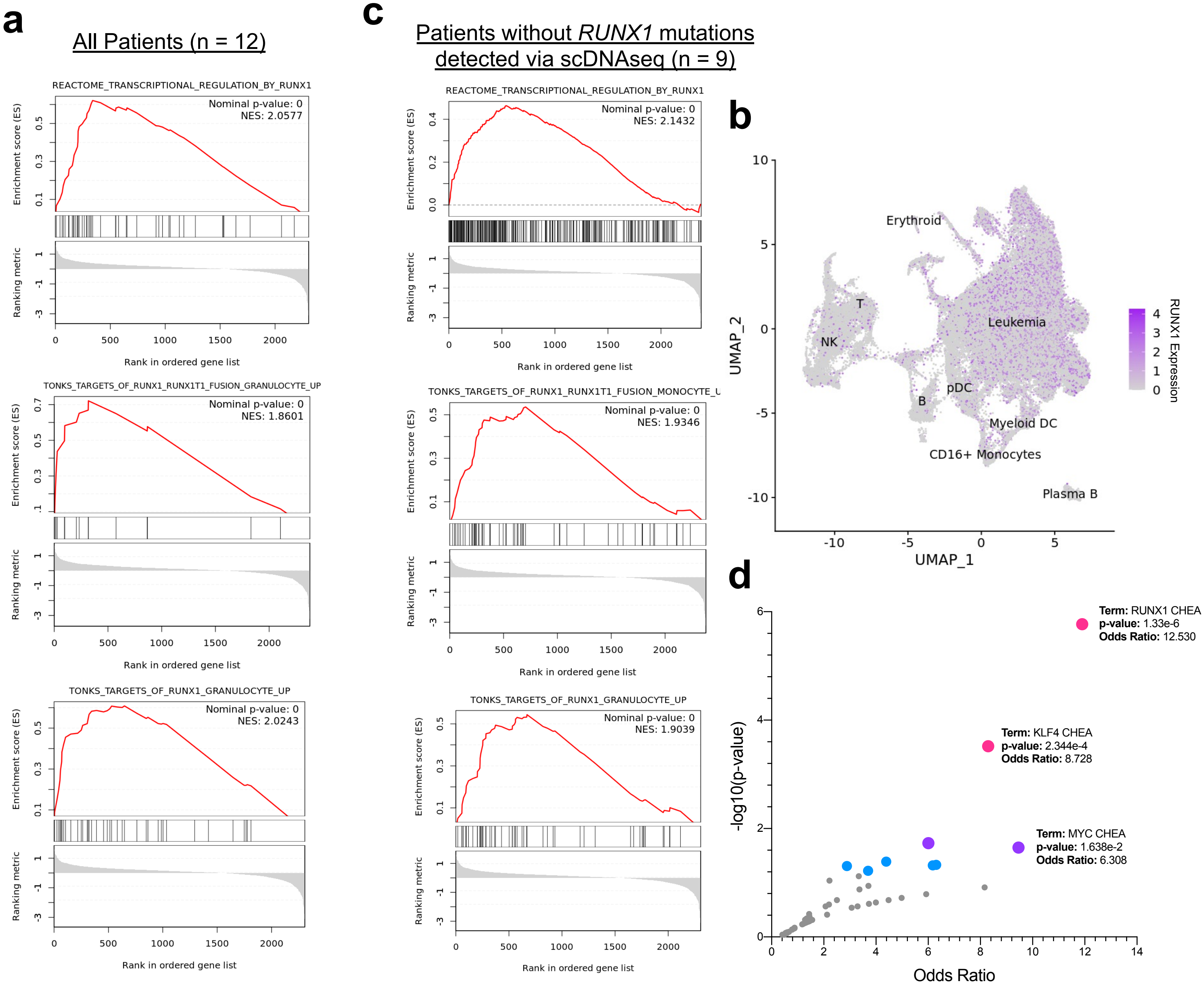

Supplementary Figure 7. The conserved gene signature associated with MPAL blasts is associated with *RUNX1* transcriptional regulation

A. Enrichment profile and ranking metric score for three gene sets associated with transcriptional regulation by *RUNX1* from GSEA (all MPAL patients, n = 12). The GSEA analysis employs a one-sided permutation-based test to determine the significance of gene set enrichment, with raw p-values adjusted for multiple testing using the Benjamini-Hochberg procedure to control the false discovery rate (FDR).

B. RNA-derived UMAP from Figure 1B, derived from single-cell RNA analysis of 71,579 cells from 12 patients with MPAL. Cells are annotated in purple based on expression of *RUNX1.*

C. Enrichment profile and ranking metric score for three gene sets associated with transcriptional regulation by *RUNX1* from GSEA (MPAL patients without known *RUNX1* mutations, n = 9). The GSEA analysis employs a one-sided permutation-based test to determine the significance of gene set enrichment, with raw p-values adjusted for multiple testing using the Benjamini-Hochberg procedure to control the false discovery rate (FDR).

D. Volcano plot of transcription factors as identified by analysis of the top differentially expressed genes in the common leukemia cluster with the ChIP-x Enrichment Analysis (ChEA) and Encyclopedia of DNA Elements (ENCODE) transcription factor targets databases via enrichr. Points color-coded based on significance as pink: $p < 0.001$, purple: $p < 0.01$, blue $p < 0.05$). The 3 most significant gene sets are annotated. (MPAL patients without known *RUNX1* mutations, n = 9). P-values are two-sided and calculated with Fisher's exact test, where genes are considered independent, and adjusted via the Benjamini-Hochberg method. Source data for all panels are provided as a Source Data file.

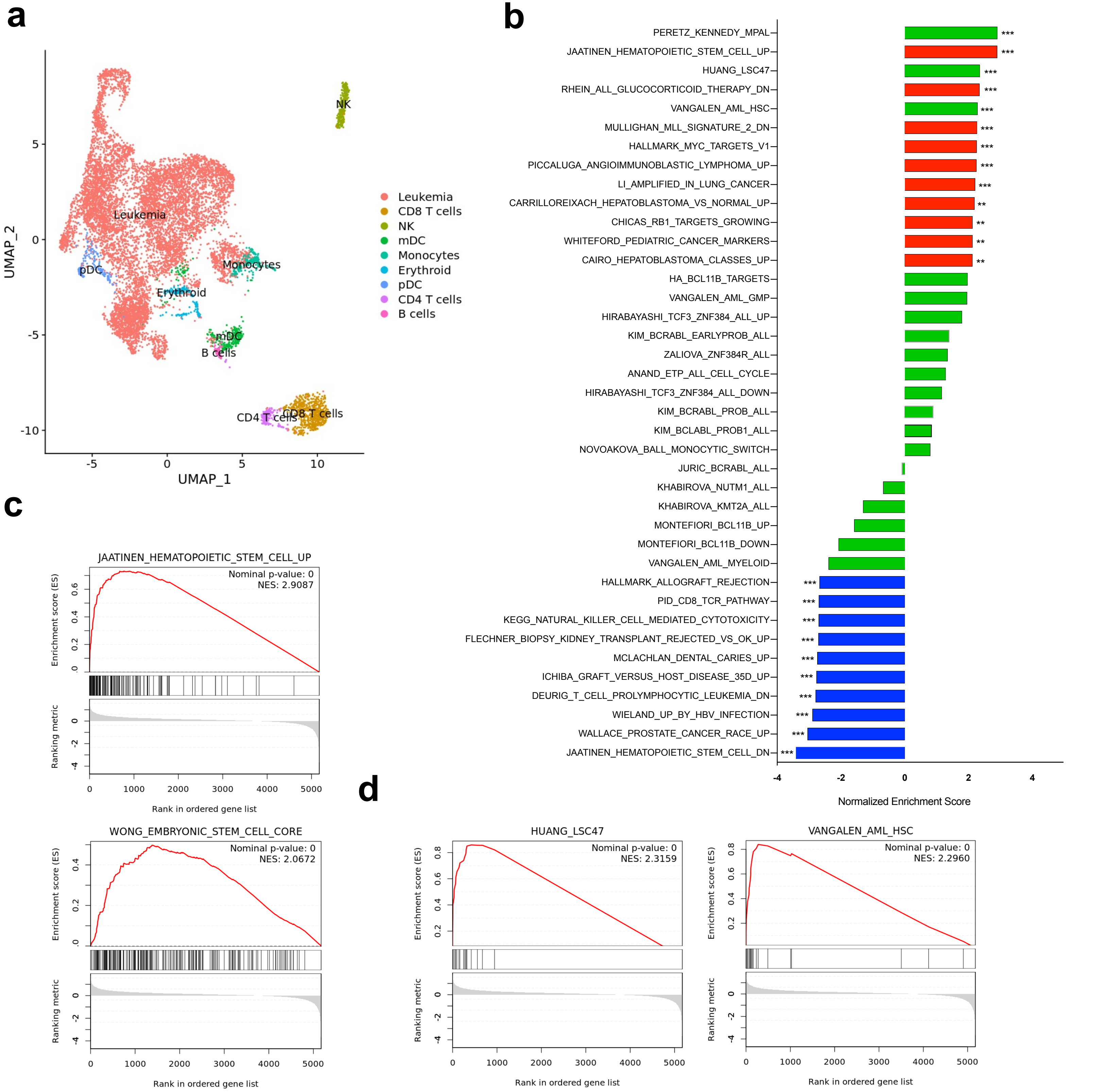

Supplementary Figure 8. An independent cohort SC-RNAseq data from 5 adult MPAL patients demonstrates greatest upregulation of the MPAL gene signature; gene sets associated with stem cells and stem-cell-like AML are significantly upregulated as well.

A. RNA-derived UMAP from SC RNAseq analysis of 11,133 cells from 5 adult patients with MPAL from an independent cohort. Cells are color-coded by cell lineage/type as determined by gene expression data.

B. Bar Plot of Normalized Enrichment Scores derived from Gene Set Expression Analysis (GSEA) of all single cells in the common leukemia cluster as identified in Figure S8A. The top 10 positively enriched gene sets are color-coded in red, the top 10 negatively enriched in blue, and additional gene sets of interest in green. Statistical significance is indicated as ***q <0.001, ** q <0.01, *q <0.05.

C. Enrichment profile and ranking metric score for two gene sets associated with stem cells.

D. Enrichment profile and ranking metric score for two gene sets associated with stem-like AML.

For B, C and D: The GSEA analysis employs a one-sided permutation-based test to determine the significance of gene set enrichment, with raw p-values adjusted for multiple testing using the Benjamini-Hochberg procedure to control the false discovery rate (FDR). Source data for all panels are provided as a Source Data file.

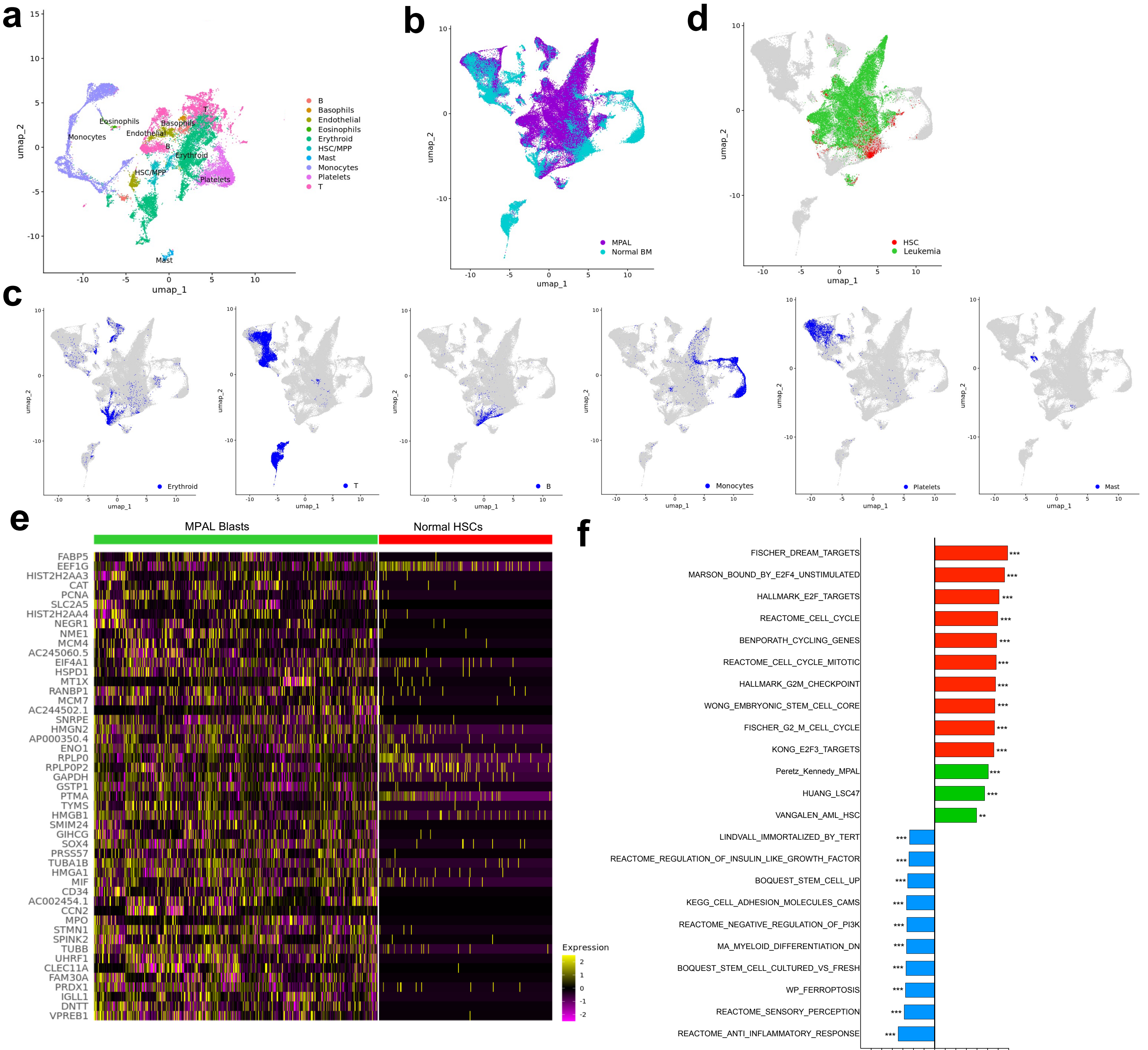

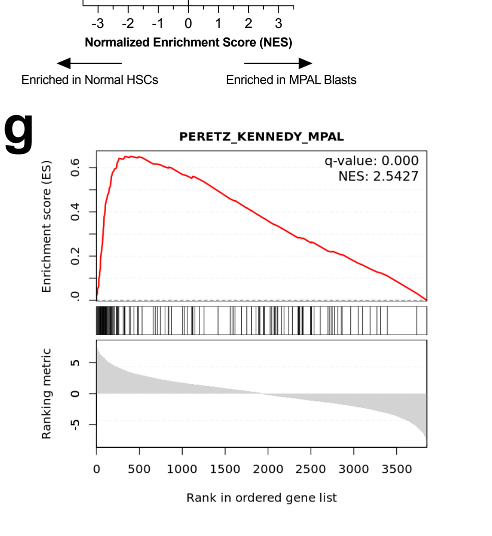

Supplementary Figure 9. MPAL blasts are transcriptionally distinct from HSCs derived from healthy donor bone marrow.

A. RNA-derived UMAP from 10,936 single cells derived from bone marrow from a healthy donor. 308 hematopoietic stem cells (HSCs) were identified via scType.

B. RNA-derived UMAP of 83,067 integrated single-cells, including 10,936 single cells from a normal bone marrow (turquoise) and 72,131 single-cells from diagnostic samples from patients with MPAL (purple).

C. Overlay of select non-leukemic cell populations on the combined and integrated UMAP, including erythroid, B, T, monocyte, platelet, and mast cell populations.

D. Overlay of HSCs (red) and MPAL blasts (green) on the combined and integrated UMAP. HSCs and MPAL blasts occupy distinct transcriptional regions.

E. Heatmap comparing gene expression of genes comprising the single-cell derived MPAL gene expression signature between MPAL blasts (left) and normal HSCs (right).

F. Bar plot of normalized enrichment score (NES) derived from Gene Set Expression Analysis (GSEA) of top 10 gene sets enriched in MPAL blasts (red) and top 10 genes enriched in normal HSCs (blue). Gene expression signatures from MPAL, the LSC47, and HSC-like AML were also assessed (green). Statistical significance is indicated as ***q <0.001, ** q <0.01, *q <0.05.

G. Enrichment profile and ranking metric score for the MPAL gene expression signature. The signature was significantly enriched in MPAL blasts relative to normal HSCs.

For F and G: The GSEA analysis employs a one-sided permutation-based test to determine the significance of gene set enrichment, with raw p-values adjusted for multiple testing using the Benjamini-Hochberg procedure to control the false discovery rate (FDR). Source data for all panels are provided as a Source Data file.

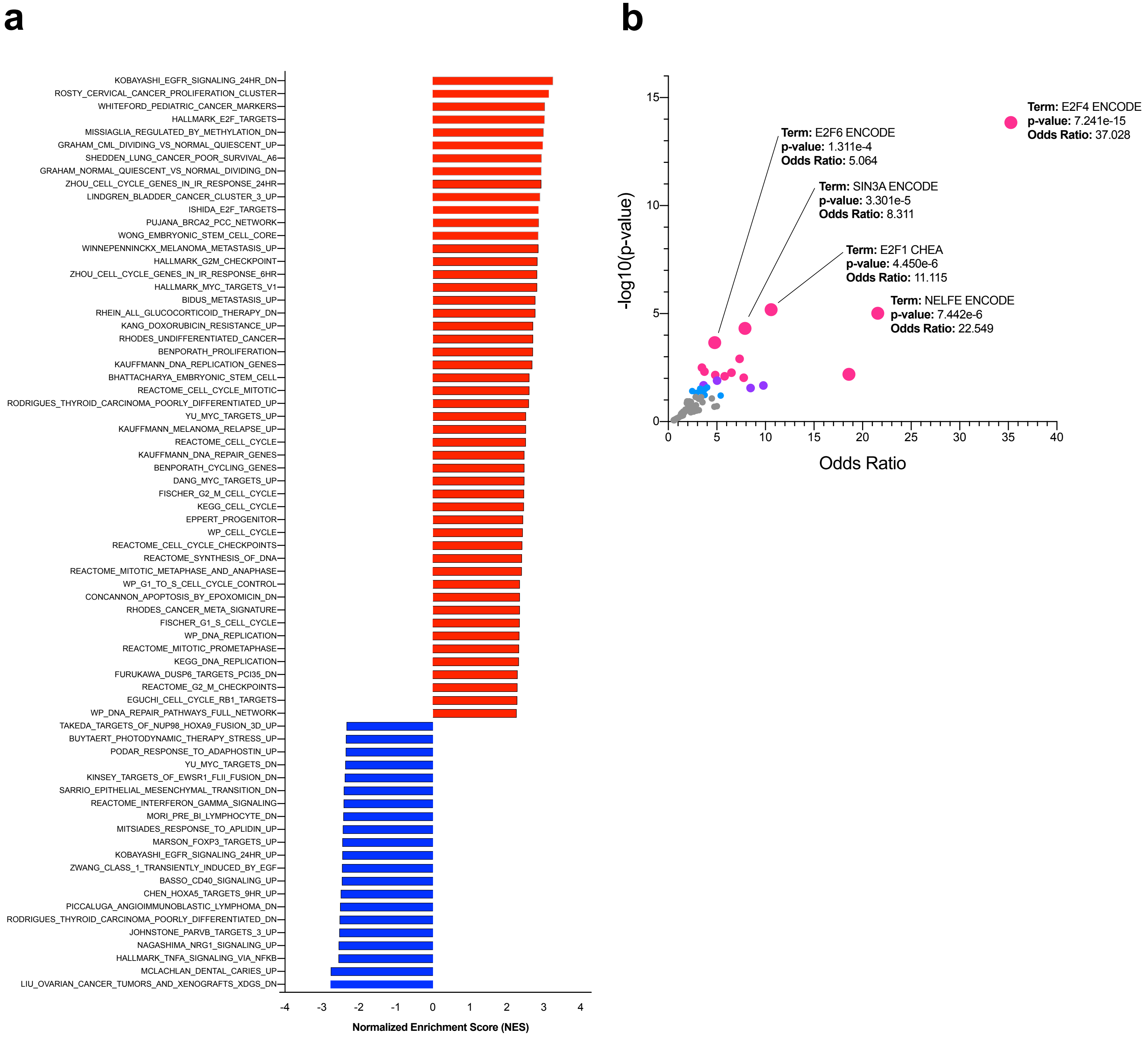

Supplementary Figure 10. Leukemia Cells with CytoTRACE >= 0.95 are associated with enrichment for pathways associated with cell division a cell cycle regulation.

A. Bar plot of normalized enrichment scores (NES) derived from Gene Set Expression Analysis (GSEA) of all leukemia cells with CytoTRACE > 0.95 vs < 0.95. Positively enriched gene sets are color-coded in red and negatively enriched gene sets are color coded in blue. All gene sets included in this figure had a statistical significance of $q < 0.001$. The GSEA analysis employs a one-sided permutation-based test to determine the significance of gene set enrichment, with raw p-values adjusted for multiple testing using the Benjamini-Hochberg procedure to control the false discovery rate (FDR).

B. Volcano plot of transcription factors as identified by analysis of the top conserved genes in the single cells with CytoTRACE >= 0.95 with the ChIP-x Enrichment Analysis (ChEA) and Encyclopedia of DNA Elements (ENCODE) transcription factor targets databases via enrichr. Points color-coded based on statistical significance as pink: $p < 0.001$, purple: $p < 0.01$, blue $p < 0.05$). The 5 most significant gene sets are annotated. P-values are two-sided and calculated with Fisher’s exact test, where genes are considered independent, and adjusted via the Benjamini-Hochberg method. Source data for all panels are provided as a Source Data file.

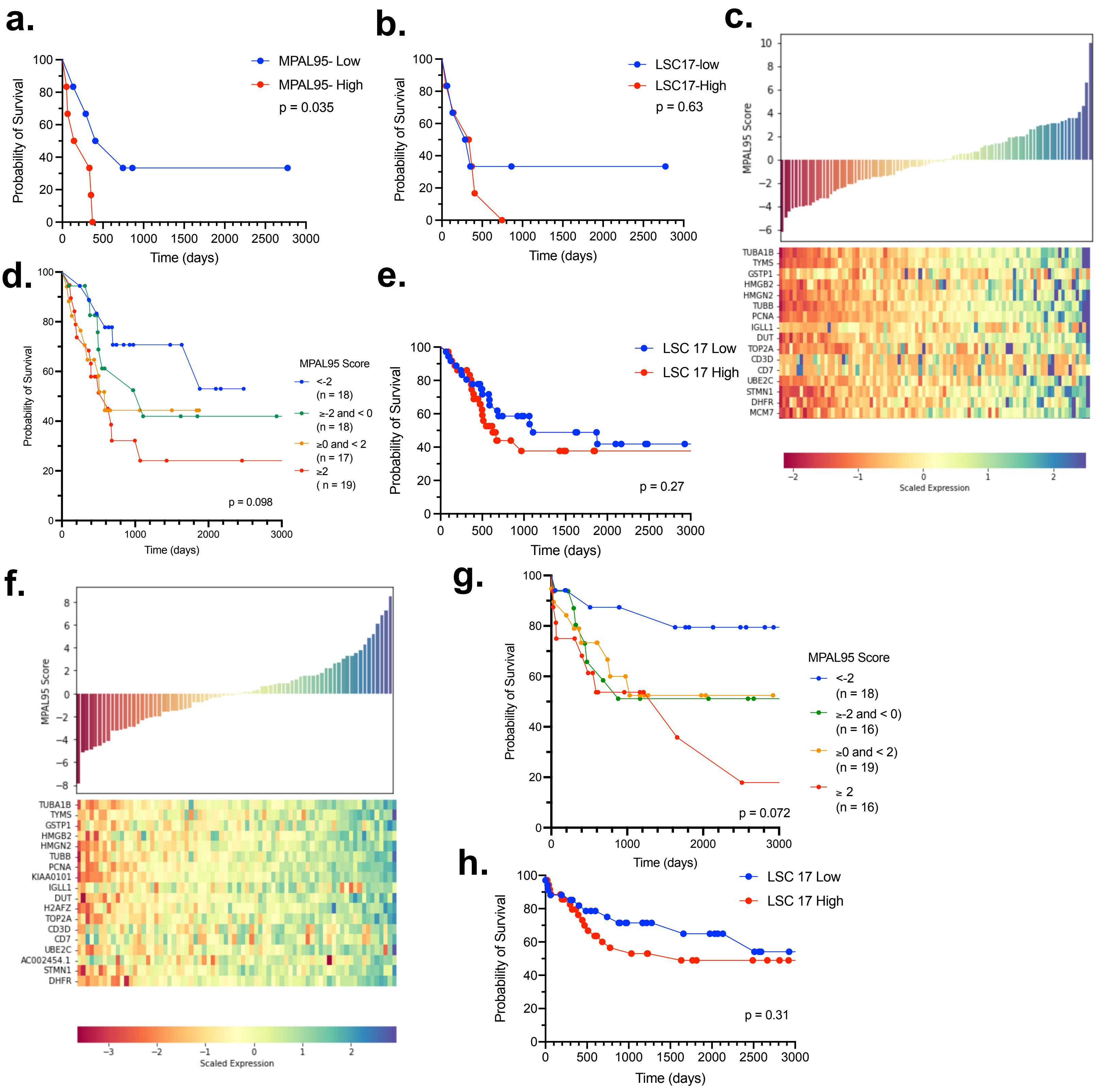

Supplementary Figure 11.
A. Kaplan Meier estimates of overall survival stratified by MPAL95 scores for pseudobulked RNAseq data from 12 adult patients with MPAL B. Kaplan Meier estimates of overall survival stratified by LSC17 scores for pseudobulked RNAseq data from 12 adult patients with MPAL C. We generated a gene set score, MPAL95, based on single-data CytoTRACE data and applied it to 69 pediatric patients with survival outcomes available from the Soochow University adult MPAL dataset (n = 72 patients). MPAL95 gene set score was computed as the first principal component (top bar plot) of the 18 genes with greatest upregulation in single cells with CytoTRACE scores ≥0.95 (bottom heatmap), where columns are individual patients. D. Kaplan Meier estimates of overall survival stratified by MPAL95 scores for Soochow University data. E. Kaplan Meier estimates of overall survival stratified by leukemia stem cell (LSC) 17 scores for the Soochow University data. F. Derivation of MPAL95 gene set score for the TARGET-ALL-P3 dataset (n = 69). G. Kaplan Meier estimates of overall survival stratified by MPAL95 scores for TARGET-ALL-P3 data. H. Kaplan Meier estimates of overall survival stratified by leukemia stem cell (LSC) 17 scores for the TARGET-ALL-P3 data. For panels A, B, D, E, G, and H, curves are compared using log-rank tests. All source data are provided as a Source Data file.

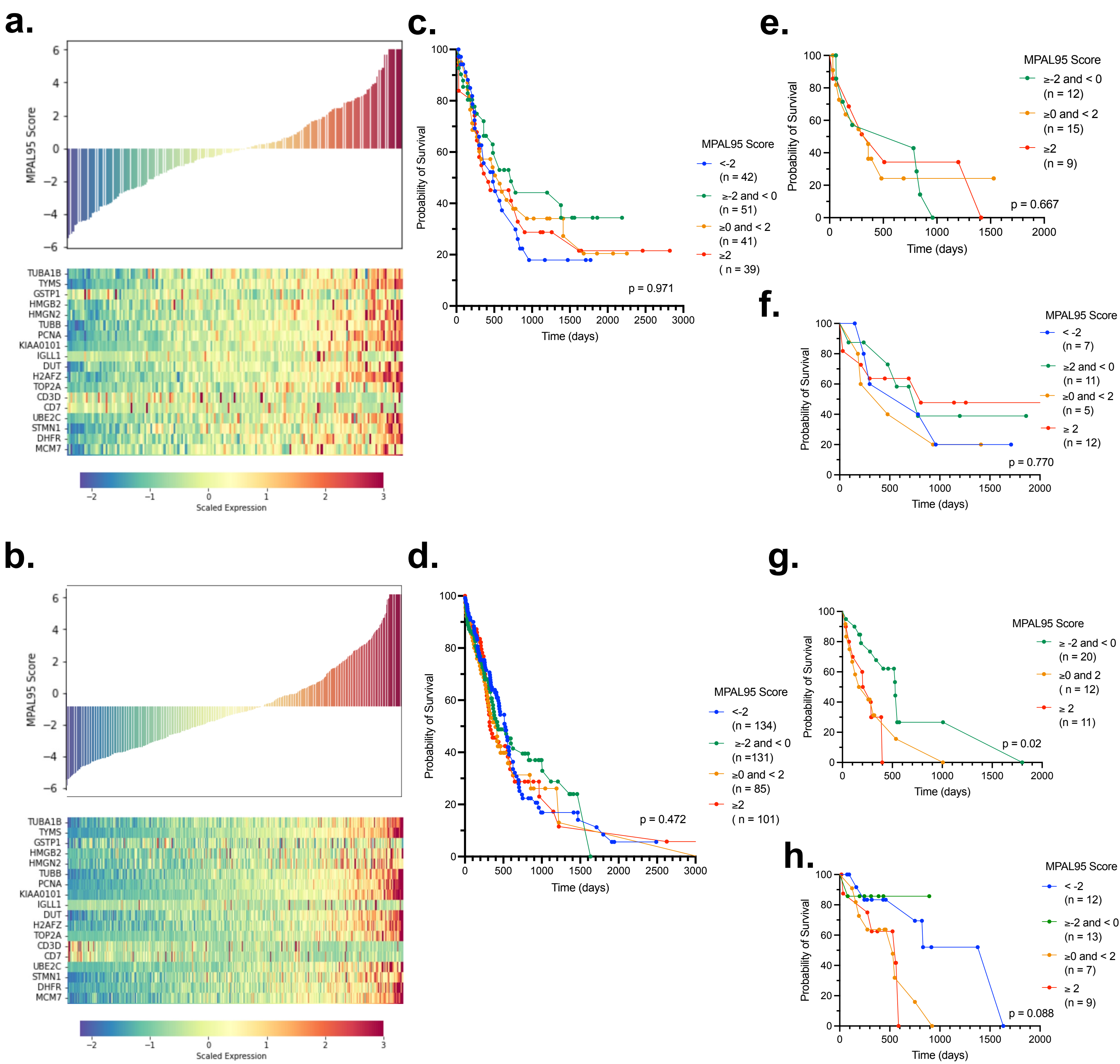

Supplementary Figure 12.

A. MPAL95 gene set score applied to 173 patients from the TCGA AML cohort. MPAL95 gene set score was computed as the first principal component (top bar plot) of the 18 genes with greatest upregulation in single cells with CytoTRACE scores ≥0.95 (bottom heatmap), where columns are individual patients.

B. MPAL95 gene set score applied to 451 patients from the Beat AML cohort.

C. Kaplan Meier estimates of overall survival stratified by MPAL95 scores for all patients in the TCGA AML cohort.

D. Kaplan Meier estimates of overall survival stratified by MPAL95 scores for all patients in the Beat AML cohort.

E. Kaplan Meier estimates of overall survival stratified by MPAL95 scores for subset of patients in the TCGA AML cohort with highest HSC-like transcriptional scores (n = 36).

F. Kaplan Meier estimates of overall survival stratified by MPAL95 scores for subset of patients in the TCGA AML cohort with highest progenitor-like transcriptional scores (n = 36).

G. Kaplan Meier estimates of overall survival stratified by MPAL95 scores for subset of patients in the Beat AML cohort with highest HSC-like transcriptional scores (n = 45**).**

H. Kaplan Meier estimates of overall survival stratified by MPAL95 scores for subset of patients in the Beat AML cohort with highest progenitor-like transcriptional scores (n = 45). For panels C-H, curves are compared using log-rank tests. Source data for all panels are provided as a Source Data file.

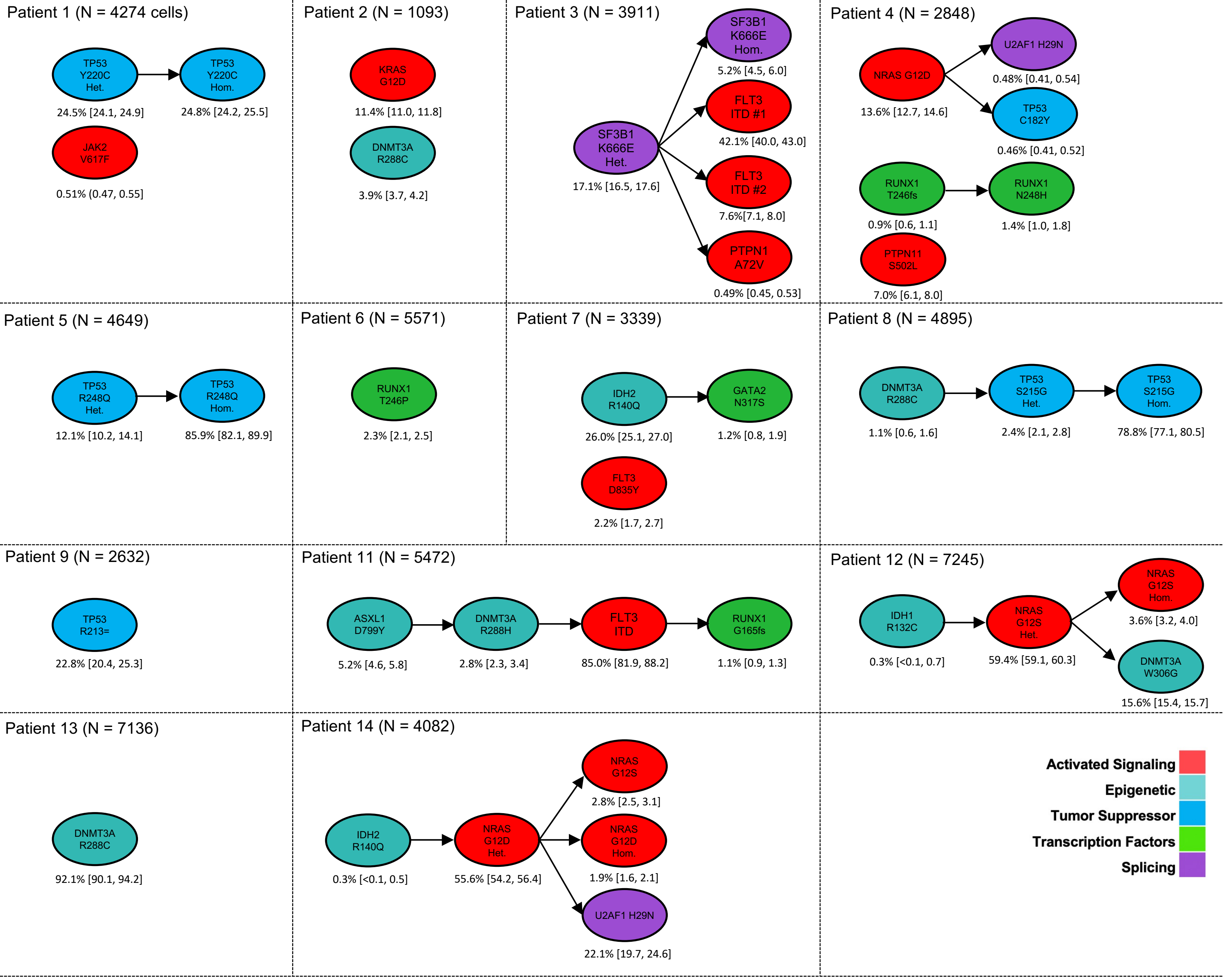

Supplementary Figure 13. Mutation phylogeny of 14 patients with newly-diagnosed MPAL derived from single-cell DNA sequencing using the SCITE algorithm. Each oval represents a subclone and arrows represent cumulative acquisition of mutational events. Subclones are color-coded based on biologic function. The percentage of each clone among the total tumor cells as well as the 95% credible intervals from the posterior sampling are shown below each oval to illustrate the uncertainty in the subclone sizes. Het: Heterozygous; Hom: Homozygous. All mutations are heterozygous unless specified otherwise. Note Patient 10 had no detectable mutations.

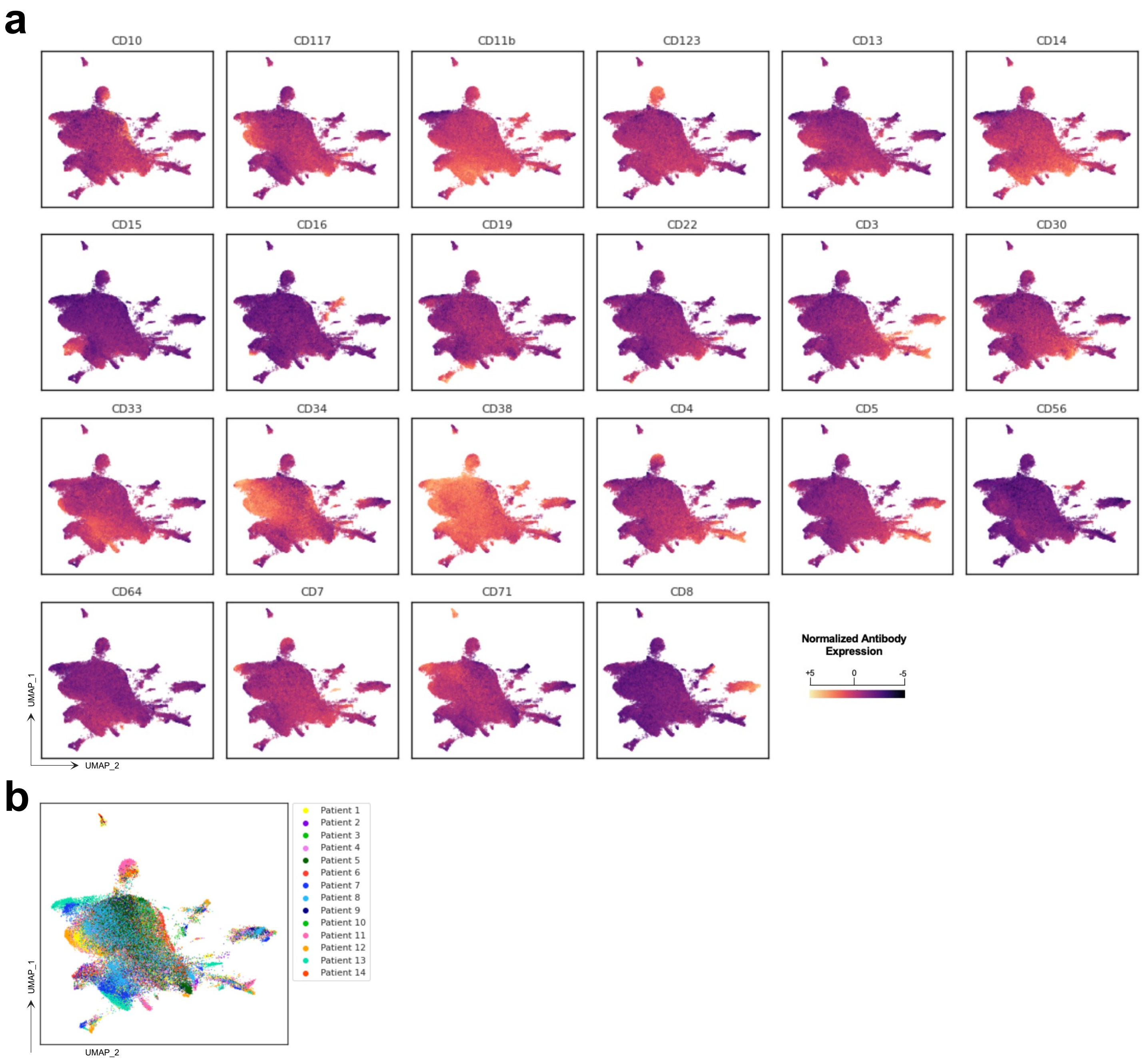

Supplementary Figure 14. Immunophenotype-derived UMAP from SC DNA+protein analysis of 51,847 cells from 14 patients (also in Figure 4F, G). Cells are color-coded based on: A. Antibody expression B. Individual patient. Source data for all panels are provided as a Source Data file.

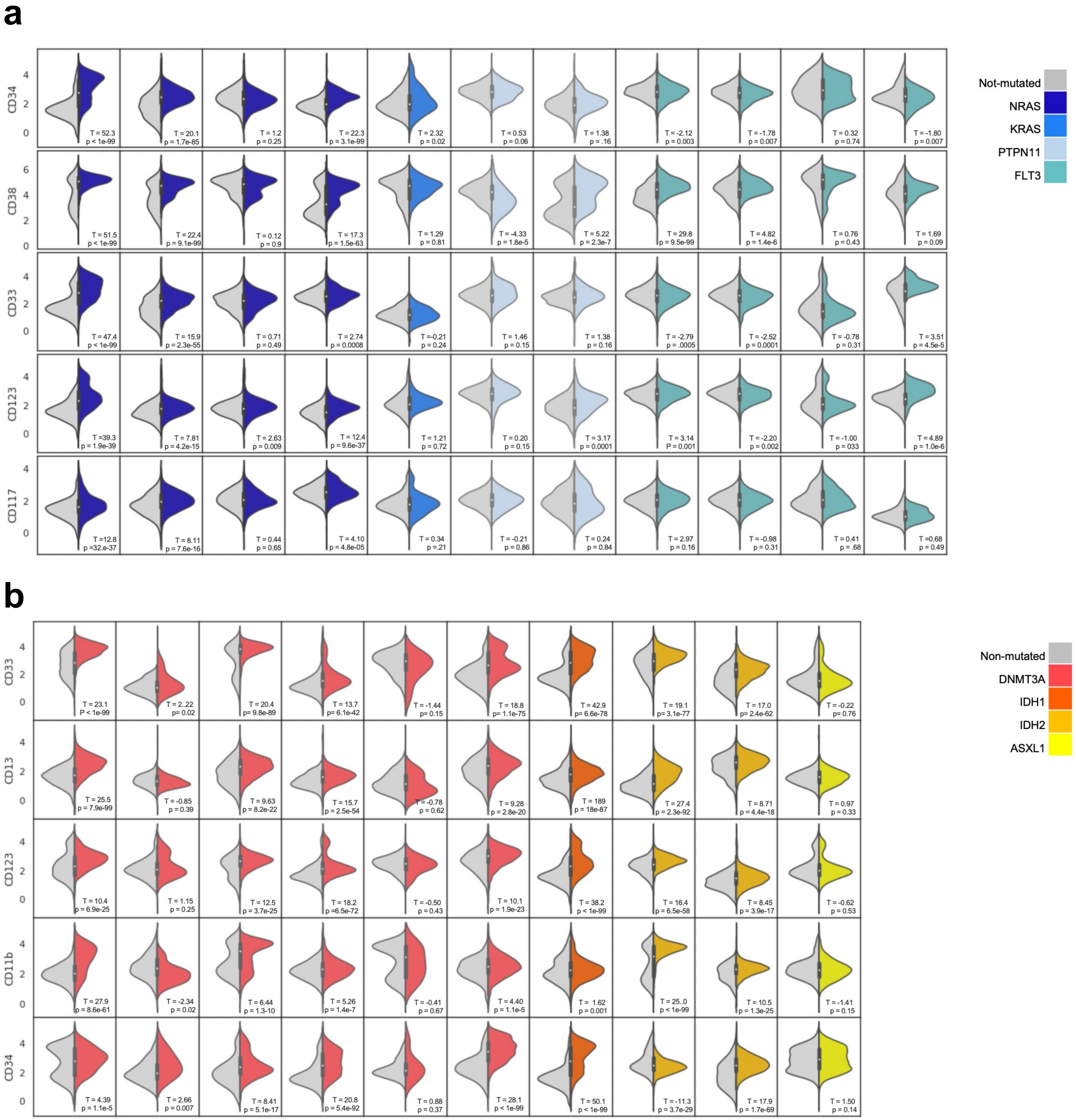

Supplementary Figure 15. Array of violin plots comparing distributions of antibody expression for mutant vs wildtype cells. **A.** Comparison of distributions of antibody expression for mutant vs wildtype cells across 11 population with signaling mutations (NRAS, KRAS, PTPN11, or FLT3). Each column represents a unique mutated population within an individual patient. Each row represents expression of 5 cell surface antibodies with the greatest median T-statistic across all 11 populations (CD34, CD38, CD33, CD123, CD117). The grey half of the split-violin plot represents non-mutated cells and the colorful half of the plot represent mutated cells within an individual patient. **B.** Comparison of distributions of antibody expression for mutant vs wildtype cells across 10 population with epigenetic modifier mutations (DNMT3A, IDH1, IDH2, ASXL1). Each column represents a unique mutated population within an individual patient. Each row represents the expression of 5 cell surface antibodies with the greatest median T-statistic across all 11 populations (CD33, CD13, CD123, CD11b,CD34). The gray half of the split-violin plot represents non-mutated cells and the colorful half of the plot represent mutated cells within an individual patient. Statistical significance is considered $p < 0.05$, with two-sided p-values calculated using Student's T test and adjusted for multiple comparisons via the Bonferroni method. Source data for all panels are provided as a Source Data file.

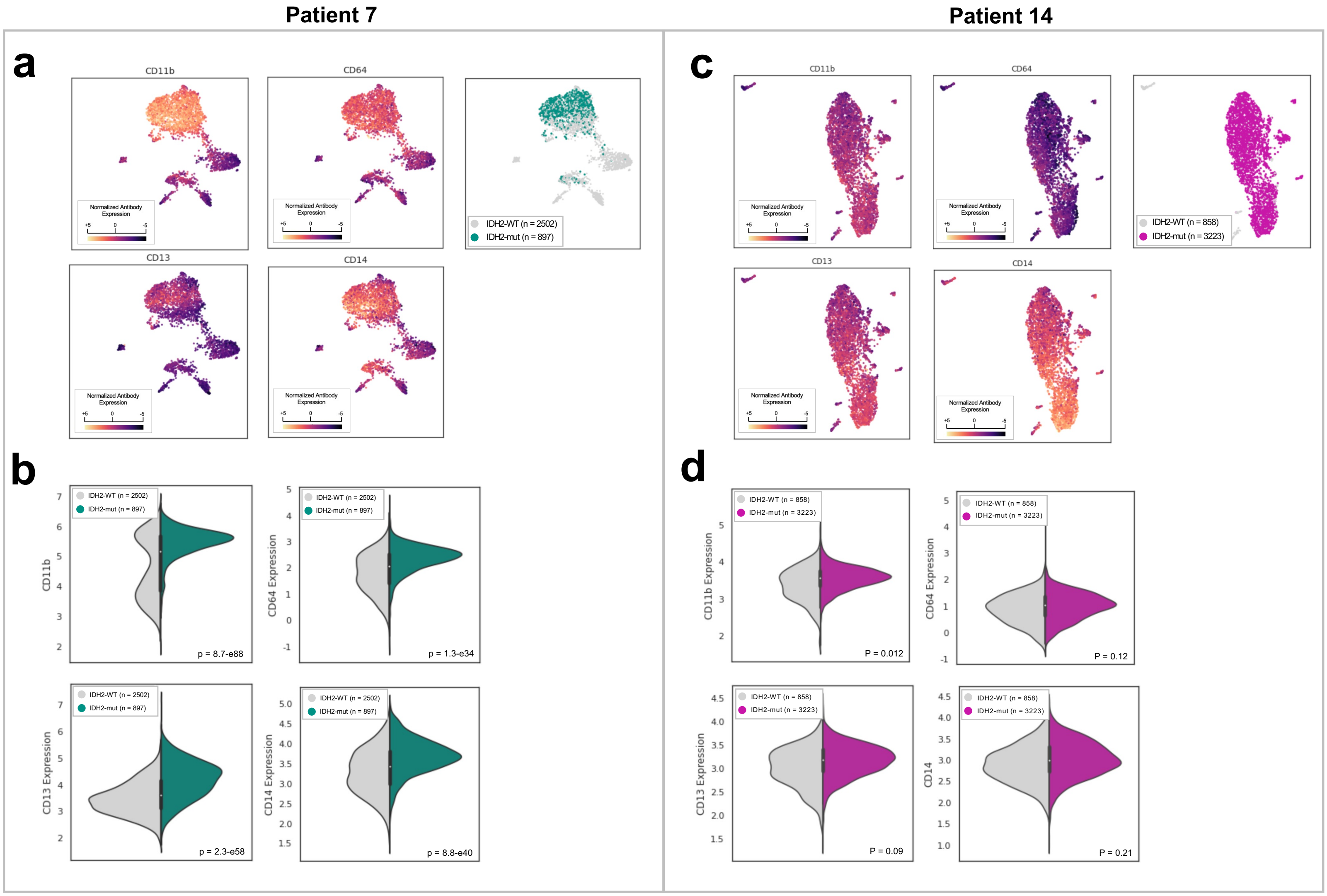

Supplementary Figure 16. Heterogenous Genotype-Immunophenotype Associations at the Patient Level are Present Even with Similar Genotypes

A..Immunophenotype-derived UMAP from 3,339 cells from **Patient 7.** Cells are color-coded based on CD11b expression (top left), CD13 expression (bottom left), CD64 expression (top center), CD14 expression (bottom center), and the presence of an IDH2 R140Q mutation (right). **B.** Violin plot comparing expression of CD11b (top left),CD13 (bottom left), CD64 (top right), and CD14 (bottom right) between IDH2-mutated vs IDH2-wildtype cells in **Patient 7. C.** Immunophenotype-derived UMAP from 4,082 cells from **Patient 14.** Cells are color-coded based on CD11b expression (top left), CD13 expression (bottom left), CD64 expression (top center), CD14 expression (bottom center), and the presence of an IDH2 R140Q mutation (right). **D.** Violin plot comparing expression of CD11b (top left),CD13 (bottom left), CD64 (top right), and CD14 (bottom right) between IDH2-mutated vs IDH2-wildtype cells in **Patient 14.** For panels B and D, statistical significance is considered $p < 0.05$, with two-sided p-values calculated using Student's T test and adjusted for multiple comparisons via the Bonferroni method. Source data for all panels are provided as a Source Data file.

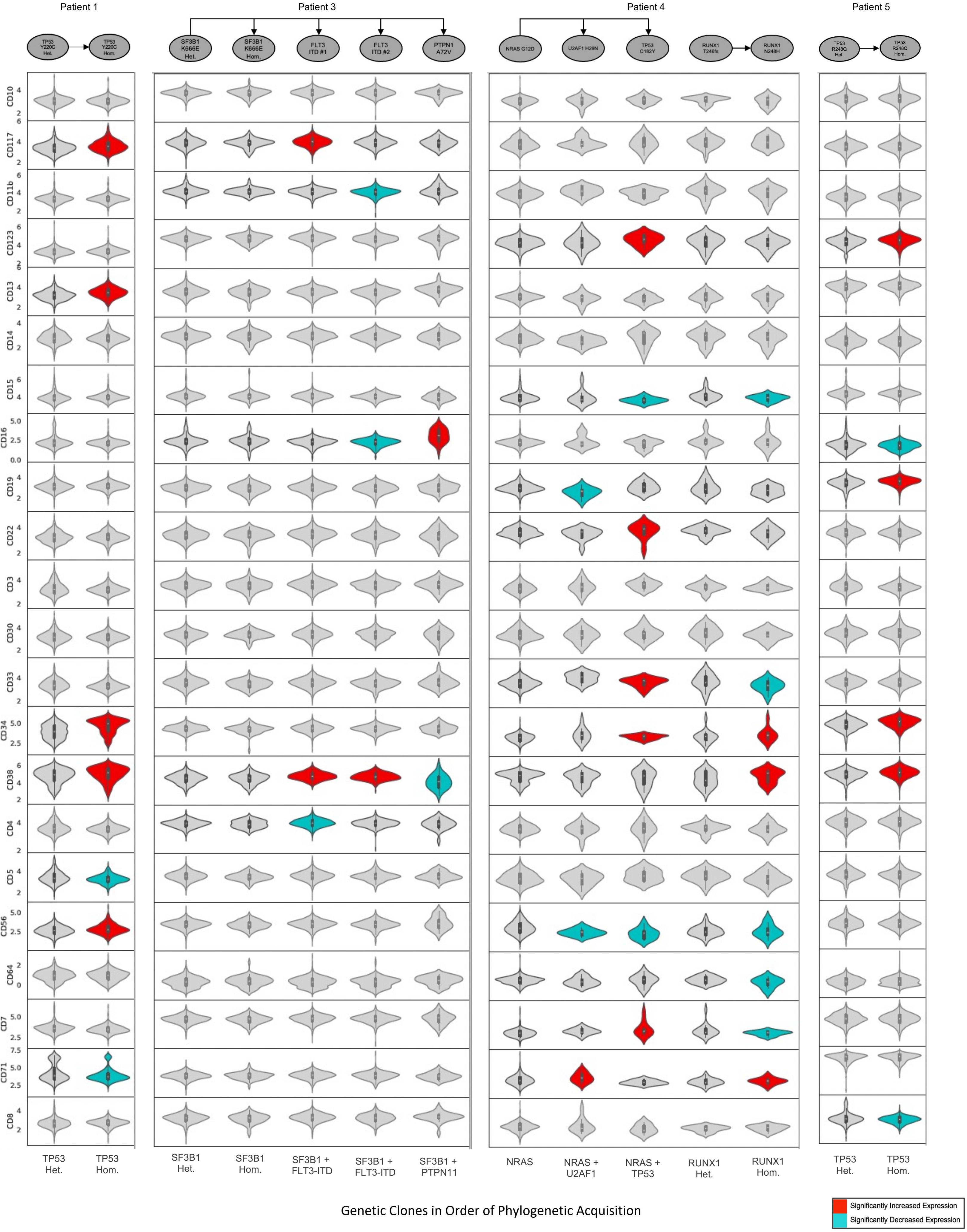

Patient 1
Patient 3
Patient 4
Patient 5
TP53 Y220C Het.
TP53 Y220C Hom.
SF3B1 K666E Het.
SF3B1 K666E Hom.
FLT3 ITD #1
FLT3 ITD #2
PTPN1 A72V
NRAS G12D
U2AF1 H29N
TP53 C182Y
RUNX1 T246fs
RUNX1 N248H
TP53 R248Q Het.
TP53 R248Q Hom.
CD10
CD117
CD11b
CD123
CD13
CD14
CD15
CD16
CD19
CD22
CD3
CD30
CD33
CD34
CD38
CD4
CD5
CD56
CD64
CD7
CD71
CD8
TP53 Het.
TP53 Hom.
SF3B1 Het.
SF3B1 Hom.
SF3B1 + FLT3-ITD
SF3B1 + FLT3-ITD
SF3B1 + PTPN11
NRAS
NRAS + U2AF1
NRAS + TP53
RUNX1 Het.
RUNX1 Hom.
TP53 Het.
TP53 Hom.
Genetic Clones in Order of Phylogenetic Acquisition
Significantly Increased Expression
Significantly Decreased Expression

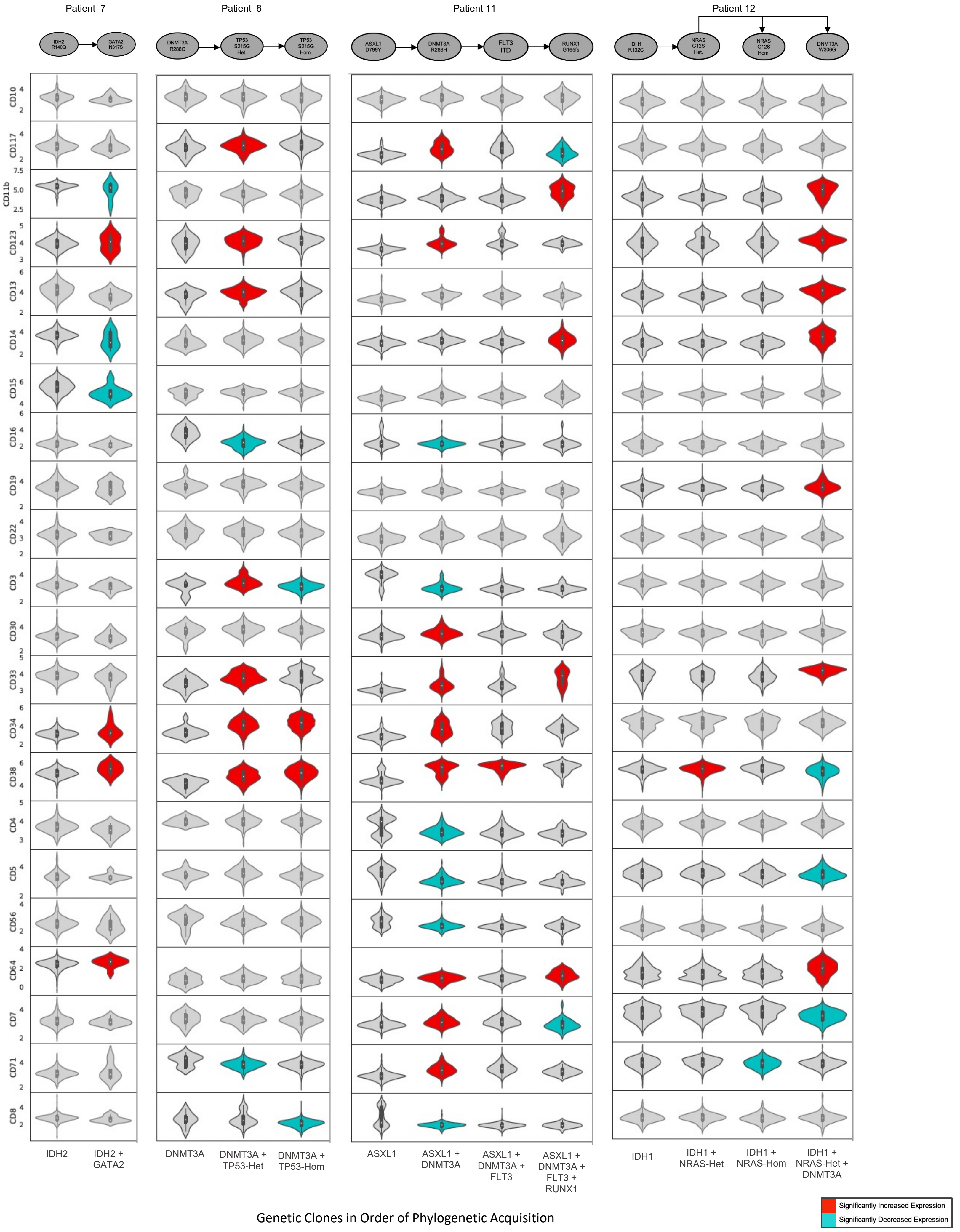

Patient 7
Patient 8
Patient 11
Patient 12
IDH2 R140Q
GATA2 N317S
DNMT3A R288C
TP53 S215G Het.
TP53 S215G Hom.
ASXL1 D799Y
DNMT3A R288H
FLT3 ITD
RUNX1 G165fs
IDH1 R132C
NRAS G12S Het.
NRAS G12S Hom.
DNMT3A W306G
CD10
CD117
CD11b
CD123
CD13
CD14
CD15
CD16
CD19
CD22
CD3
CD30
CD33
CD34
CD38
CD4
CD5
CD56
CD64
CD7
CD71
CD8
IDH2
IDH2 + GATA2
DNMT3A
DNMT3A + TP53-Het
DNMT3A + TP53-Hom
ASXL1
ASXL1 + DNMT3A
ASXL1 + DNMT3A + FLT3
ASXL1 + DNMT3A + FLT3 + RUNX1
IDH1
IDH1 + NRAS-Het
IDH1 + NRAS-Hom
IDH1 + NRAS-Het + DNMT3A
Genetic Clones in Order of Phylogenetic Acquisition
Significantly Increased Expression
Significantly Decreased Expression

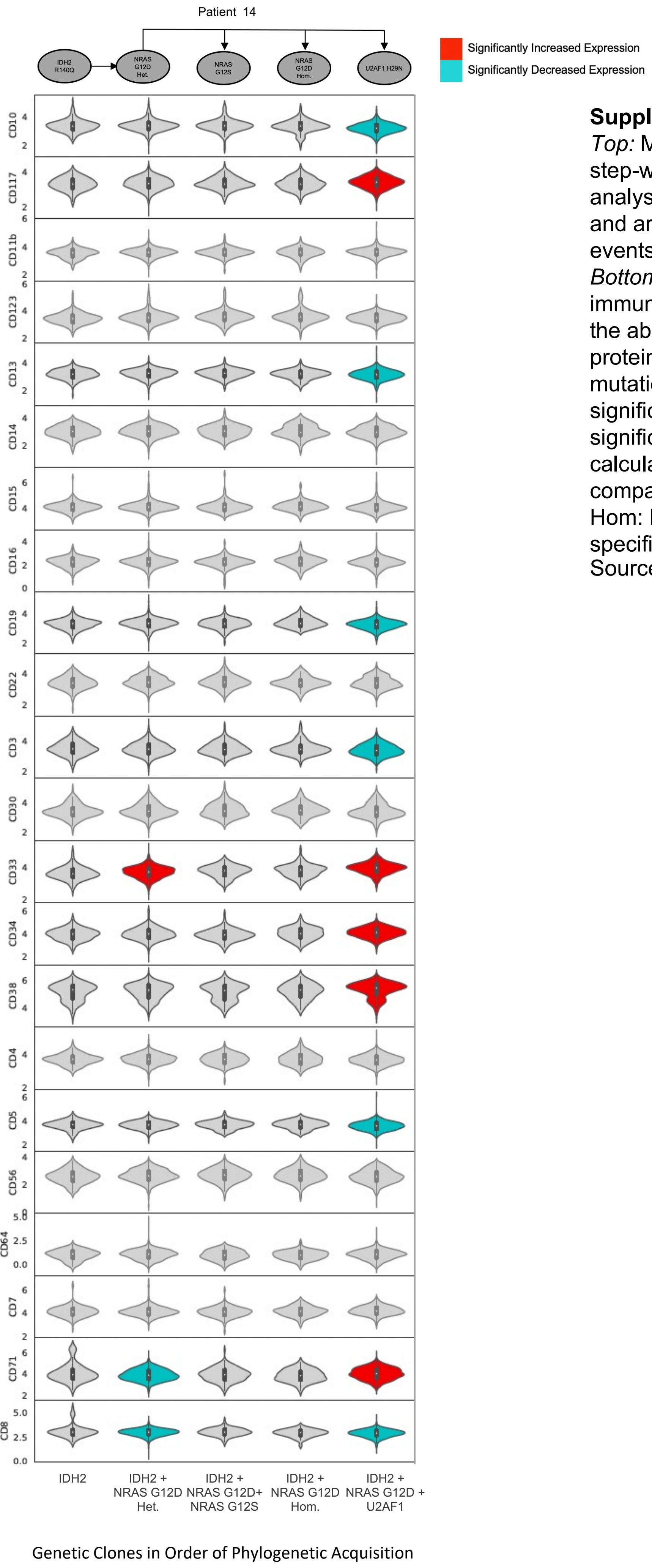

Supplementary Figure 17.
Top: Mutation phylogeny of 9 patients with MPAL with at least 2 step-wise mutational acquisitions identified on single-cell DNA analysis. Each oval represents a genetically-distinct subclone and arrows represent cumulative acquisition of mutational events.
Bottom: Violin plots depicting expression of 22 immunophenotypic proteins for each subclone represented in the above phylogeny. Violin plots color-coded in red indicate protein expression that has significantly increased with mutational acquisition; plots color-coded in blue indicate a significant decrease in protein-expression. Statistical significance is considered $p < 0.05$, with two-sided p-values calculated using Student's T test and adjusted for multiple comparisons via the Bonferroni method. Het: Heterozygous; Hom: Homozygous. All mutations are heterozygous unless specified otherwise.
Source data are provided as a Source Data file.
