# Supplementary material for: Multiomic single cell sequencing identifies stemlike nature of mixed phenotype acute leukemia
Source: Nat Commun. 2024 Sep 18;15:8191. doi: 10.1038/s41467-024-52317-2 (PMC11411136; doi:10.1038/s41467-024-52317-2)
Supplement: Supplementary file 3 — Description of Additional Supplementary Files [file 41467_2024_52317_MOESM3_ESM.pdf]

Description of Additional Supplementary Files

Supplementary Data 1: Cinical Characteristics of 14 Adult Patients with Newly-Diagnosed MPAL

Supplementary Data 2: Number of Single Cells Derived from Dabseq and CITEseq single cell assays

Supplementary Data 3: Antibody-Derived Tags (ADT) used in single-cell CITE-seq (2.5 ug/mL)

Supplementary Data 4: Top Expressed Genes for Each Cell Type Ranked by Average log2FC

Supplementary Data 5: Expression of 12 conserved genes from the common leukemia cluster by patient

Supplementary Data 6: Gene Set Enrichment Analysis (GSEA) Results for single cell in the common leukemia cluster

Supplementary Data 7: Additional Previously-Published Gene Sets Associated with Immature and Mature AML, ALL, ALL with subsequent monocytic switch, and gene rearrangements characteristic of MPAL

Supplementary Data 8: Top Upregulared Genes in Common Leukemia Cluster of Single-Cell Cohort ("Peretz_Kennedy_MPAL") and Genes Comprising Single Cells with Highest CytoTRACE vaues ("MPAL95")

Supplementary Data 9: Patient and Disease Characteristics of the 69 Pediatric MPAL Patients with Survival Data Available from the TARGET Cohort

Supplementary Data 10: Targeted DNA Panel Used in Single-Cell DAb-seq

Supplementary Data 11: Antibody-Oligo Conjugates (AOCs) for SC DNA+Protein sequencing

Supplementary Data 12: All Pathogenic or Likely Pathogenic Mutations as Identified by Single Cell DAb-seq
