# Supplementary material for: Multiomic single cell sequencing identifies stemlike nature of mixed phenotype acute leukemia
Source: Nat Commun. 2024 Sep 18;15:8191. doi: 10.1038/s41467-024-52317-2 (PMC11411136; doi:10.1038/s41467-024-52317-2)
Supplement: Supplementary file 16 — Reporting Summary [file 41467_2024_52317_MOESM16_ESM.pdf]

nature portfolio

Corresponding author(s): Catherine Smith

Last updated by author(s): Jun 24, 2024

Reporting Summary

Nature Portfolio wishes to improve the reproducibility of the work that we publish. This form provides structure for consistency and transparency in reporting. For further information on Nature Portfolio policies, see our Editorial Policies and the Editorial Policy Checklist.

Statistics

For all statistical analyses, confirm that the following items are present in the figure legend, table legend, main text, or Methods section.

n/a	Confirmed	
☐	☒	The exact sample size (*n*) for each experimental group/condition, given as a discrete number and unit of measurement
☐	☒	A statement on whether measurements were taken from distinct samples or whether the same sample was measured repeatedly
☐	☒	The statistical test(s) used AND whether they are one- or two-sided<br>*Only common tests should be described solely by name; describe more complex techniques in the Methods section.*
☐	☒	A description of all covariates tested
☐	☒	A description of any assumptions or corrections, such as tests of normality and adjustment for multiple comparisons
☐	☒	A full description of the statistical parameters including central tendency (e.g. means) or other basic estimates (e.g. regression coefficient) AND variation (e.g. standard deviation) or associated estimates of uncertainty (e.g. confidence intervals)
☐	☒	For null hypothesis testing, the test statistic (e.g. *F*, *t*, *r*) with confidence intervals, effect sizes, degrees of freedom and *P* value noted<br>*Give P values as exact values whenever suitable.*
☐	☒	For Bayesian analysis, information on the choice of priors and Markov chain Monte Carlo settings
☒	☐	For hierarchical and complex designs, identification of the appropriate level for tests and full reporting of outcomes
☒	☐	Estimates of effect sizes (e.g. Cohen's *d*, Pearson's *r*), indicating how they were calculated

Our web collection on statistics for biologists contains articles on many of the points above.

Software and code

Policy information about availability of computer code

Data collection	Raw fastqs for single-cell RNA-seq data were processed using the commercial pipeline PIPseeker v0.52 (Fluent Biosciences). Raw fastqs for single-cell DAb-seq data was processed using an open-source pipeline (https://github.com/AbateLab/DAb-seq; git pull on 8/1/2021). For the open-source DAb-seq pipeline, the following dependencies were used: GATK (v4.1.3.0), bowtie2 (.2.3.4.1), ITDseek (v1.2), samtools (v1.8), bedtooks (v2.27.1) bcftools (v1.9), cutadapt (v2.4), BBMap (v38.57), snpEff (v4.3t). The ClinVar database (v.20190805) was used for variant annotation.
Data analysis	Single-cell RNA-seq data was analyzed using R (version 4.0.2) with the open-source Seurat package (version 4.3.0). Additional downstream RNA-seq analyses were conducted using gsea (version 4.2.3), CytoTRACE (version 0.3.3), and custom code (https://github.com/SmithLabUCSF/MPAL). Single-cell DAbseq data was analyzed using custom code (https://github.com/SmithLabUCSF/MPAL), with the following packages: pandas (v1.0.3), numpy (v1.18.1), umap v0.3.10), anndata (v.0.7.1), scikit-learn (v0.22.1). All statistical analyses were performed in R (v. 4.0.2). Downstream analysis scripts are available at github.com/SmithLabUCSF/MPAL.

For manuscripts utilizing custom algorithms or software that are central to the research but not yet described in published literature, software must be made available to editors and reviewers. We strongly encourage code deposition in a community repository (e.g. GitHub). See the Nature Portfolio guidelines for submitting code & software for further information.

Data

Policy information about availability of data

All manuscripts must include a data availability statement. This statement should provide the following information, where applicable:

- Accession codes, unique identifiers, or web links for publicly available datasets
- A description of any restrictions on data availability
- For clinical datasets or third party data, please ensure that the statement adheres to our policy

The data as generated here, including raw sequencing data in the form of FASTQ files, have been deposited in NCBI's Gene Expression Omnibus36 (GEO) and are accessible through GEO series accession number GSE232074 [https://www.ncbi.nlm.nih.gov/geo/query/acc.cgi?acc=GSE232074]. Comparison cohorts include single cell RNAseq data from a cohort of 5 adult patients with MPAL28 deposited in GEO and accessible through GEO Accession Code GSE139369 [https://www.ncbi.nlm.nih.gov/geo/query/acc.cgi?acc=GSE139369], bulk RNAseq data from the pediatric database "Therapeutically Applicable Research to Generate Effective Treatments (TARGET)" initiative43 which is publicly available through [https://ocg.cancer.gov/programs/target/projects/acute-lymphoblastic-leukemia2021.:], and bulk RNAseq data from a cohort of adult patients treated at First Affiliated Hospital of Soochow University, Suzhou, China, which was requested directly from corresponding authors42. All source data are provided with this paper.

Research involving human participants, their data, or biological material

Policy information about studies with human participants or human data. See also policy information about sex, gender (identity/presentation), and sexual orientation and race, ethnicity and racism.

Reporting on sex and gender	Our single-cell cohort included 5 male and 9 female patients based on biologic sex as reported in the patient's electronic medical record and accessed by the study authors via a de-identified database. Patient-level sex is indicated in Supplementary Data 1, and patients provided consent for de-identified sex data to be included as part of the consent for tissue banking at their treating institution. All adult patients with a diagnosis of Mixed Phenotypic Acute Leukemia and a peripheral blood or bone marrow sample of viable cells banked in the University of California San Francisco or University of Pennsylvania tissue bank were included in this study; neither patient sex nor gender were considered in study design. No sex-based analyses was performed due to the relatively small cohort size.
Reporting on race, ethnicity, or other socially relevant groupings	This clinical information was not available to us as part of our de-identified data set.
Population characteristics	Patient age, treatment information, clinical response to treatment, and genotypic information is detailed in Supplementary Table 1.
Recruitment	All adult patients with a diagnosis of Mixed Phenotypic Acute Leukemia and a peripheral blood or bone marrow sample of viable cells banked in the University of California San Francisco or University of Pennsylvania tissue bank were included in this study. Due to various socioeconomic factors, some patient demographics are unlikely to be treated at academic medical centers and/or are unlikely to sign consent for tissue banking for research purposes; these patient demographics will not be represented in this study and thus may bias our results.
Ethics oversight	All patients provided written informed consent for sample banking and analysis under protocols approved by the local Institutional Review Board (either the University of California San Francisco or the University of Pennsylvania) and this research was conducted in accordance with the ethical standard of the institution and with the Declaration of Helsinki.

Note that full information on the approval of the study protocol must also be provided in the manuscript.

Field-specific reporting

Please select the one below that is the best fit for your research. If you are not sure, read the appropriate sections before making your selection.

☒ Life sciences ☐ Behavioural & social sciences ☐ Ecological, evolutionary & environmental sciences

For a reference copy of the document with all sections, see nature.com/documents/nr-reporting-summary-flat.pdf

Life sciences study design

All studies must disclose on these points even when the disclosure is negative.

Sample size	All adult patients with a diagnosis of Mixed Phenotypic Acute Leukemia (MPAL) and a peripheral blood or bone marrow sample of viable cells banked in the University of California San Francisco or University of Pennsylvania tissue bank were included in this study, resulting in a cohort of 14 patients. All 14 patients were included in single-cell DAb-seq analysis; due to inadequate cell thawing, 12 of 14 patients were including in single-cell PIP-seq analysis. No statistical methods were used to determine this sample size. As MPAL is a rare leukemia, we chose to include all available samples, and this represents the largest cohort of adult patients with MPAL in single-cell analysis to date. On the sample level, single-cell DAb-seq and PIP-seq were performed on 1,000 - 10,000 cells, consistent with sample sizes used by others in the field.
Data exclusions	Two patients in our original cohort of 14 patients were not included in PIP-seq analysis due to inadequate thawing of the frozen sample. All

Data exclusions	patients for whom single-cell analysis was complete were included without exclusion.
Replication	Our single-cell DAb-seq and PIP-seq analyses did not have biological replicates due to the limited number of frozen viable cells available.
Randomization	Randomization was not relevant to this study. All patient samples were processed and analyzed identically, regardless of patient or disease characteristics.
Blinding	Blinding was not relevant to this study. All patient samples were processed and analyzed identically, regardless of patient or disease characteristics. Patient-level identifiers were used to associate PIP-seq analyses, including medium CytoTRACE score, with patient survival following the completion of all single-cell processing and analysis.

Reporting for specific materials, systems and methods

We require information from authors about some types of materials, experimental systems and methods used in many studies. Here, indicate whether each material, system or method listed is relevant to your study. If you are not sure if a list item applies to your research, read the appropriate section before selecting a response.

Materials & experimental systems

n/a	Involved in the study
☐	☒ Antibodies
☒	☐ Eukaryotic cell lines
☒	☐ Palaeontology and archaeology
☒	☐ Animals and other organisms
☐	☒ Clinical data
☒	☐ Dual use research of concern
☒	☐ Plants

Methods

n/a	Involved in the study
☒	☐ ChIP-seq
☒	☐ Flow cytometry
☒	☐ MRI-based neuroimaging

Antibodies

Antibodies used	Please see Supplementary Data 11 for the product name, barcode, clone and supplier link for all antibody-oligo conjugates used in single-cell DAb-seq. Antibodies used at 2.5 ug/mL. Please see Supplementary Data 3 for the product name, barcode, clone, and catalog number for all antibody-oligo conjugates used in single-cell PIP-seq. Antibodies used at 10ug/mL. Each antibody with mulitple citations as per BioLegend website https://www.biolegend.com/de-de/products/
Validation	For DAb-seq analysis, experimental validation data and quality certificates are provided on the commercial supplier's website as listed in Supplementary Data 3. For PIP-seq analysis, all antibodies were validated by BioLegend (https://biolegend.com/nl-nl/reproducibility).

Clinical data

Policy information about clinical studies

All manuscripts should comply with the ICMJE guidelines for publication of clinical research and a completed CONSORT checklist must be included with all submissions.

Clinical trial registration	The patient participants in this study were not enrolled on a clinical trial.
Study protocol	The patient participants in this data were not enrolled on a study protocol.
Data collection	All patients were diagnosed with diagnostic peripheral blood or bone marrow samples banked between 2006 -2020. Sample preparation, sequencing, and analysis occurred between June 2021 - January 2023.
Outcomes	Specific clinical outcomes were not measured as part of this study. Survival outcomes, including last available follow up time and patient status (alive or dead) at last follow up were obtained a de-identified clinical databased linked to the patient's electronic medical record.

Plants

Seed stocks	N/A
Novel plant genotypes	N/A
Authentication	N/A
